# Optimization of skeleton structure discrimination standard for SMA-13 mixture based on high-temperature performance

**DOI:** 10.1371/journal.pone.0331954

**Published:** 2025-09-17

**Authors:** Jinshun Xue, Xiao Chen, Wei Zhao, Yuanyuan Wang

**Affiliations:** 1 Hubei Key Laboratory of Vehicle-infrastructure Cooperation and Traffic Control, Xiangyang, China; 2 School of Civil Engineering and Architecture, Hubei University of Arts and Science, Xiangyang, China; 3 HPCPDI Ruizhi Transportation Technology Consulting Co., Ltd., Shijiazhuang, China; 4 Hebei Province Road Structure and Material Technology Innovation Center Shijiazhuang, China; 5 School of Civil Engineering and Architecture, Hubei University of Arts and Science, Xiangyang, China; Shandong University of Technology, CHINA

## Abstract

The stone–on–stone contact-interlocked skeleton structure of stone mastic asphalt (SMA) mixtures is crucial for the effective design of the mixture. However, existing skeleton structure discrimination standard (*VCA*_mix_ < *VCA*_DRC_) for SMA mixture gradation design is proven to necessitate calibration. To address this, a method was developed to analyse the influence of structural parameters on the high-temperature performance of SMA-13 mixture. And a skeleton structure discrimination standard for the SMA mixture was proposed and subsequently verified through the high-temperature performance of the SMA-13 mixture. The results indicate that variations in testing methods, compaction efforts, and coarse aggregate breakage lead to discrepancies between the key parameters *VCA*_DRC_ and *VCA*_mix_ when evaluating the skeleton structure discrimination standard for SMA mixtures. Therefore, a volume method was introduced to the *VCA*_mix_ expression, calculating it using the volume of the cylindrical specimen compacted by the VTM method. Additionally, the *VCA*_mix_ < 0.95*VCA*_DRC(n=25)_ is recommended as the skeleton structure discrimination standard for SMA mixture gradation design. Which exhibited more prominent high-temperature performance (8% higher shear strength and 15% greater dynamic stability) than the SMA mixtures designed using *VCA*_mix_ < *VCA*_DRC(n=25)_ as the skeleton structure discrimination standard.

## 1. Introduction

Stone mastic asphalt (SMA) mixtures, first introduced in Europe in the 1960s, are now used globally owing to their excellent high-temperature rutting resistance and durability [1–2]. SMA mixtures primarily consist of coarse and fine aggregates, mineral powder, asphalt, and fiber stabilizers [3]. They exhibit a “three more and less” compositional structure [4], characterized by higher contents of coarse aggregate (70–80%) [5], mineral powder, asphalt, and a lower content of fine aggregate. The coarse aggregate forms a stone–stone contact interlocked skeleton structure, which bestows the SMA mixture with superior resistance to rutting under high-temperature loading conditions [6]. The performance of densely graded asphalt and SMA mixtures was compared by Asi [7] based on compressive and tensile strengths, durability, and resilience properties. The results indicated that SMA mixtures outperform densely graded mixtures in hot climate conditions. SMA mixtures also exhibit excellent resistance to freeze–thaw damage [8]. Additionally, the content and characteristics of coarse aggregates significantly influence the skeleton structure of SMA mixtures, which, in turn, affect pavement performance. The rut-depth of SMA mixtures increases with higher sphericity and decreases with greater angularity and surface texture of the coarse aggregates. This suggests that flat and elongated aggregates should be used only in limited amounts, whereas more equi-dimensional, angular aggregates with rough textures should be preferred to improve the rutting resistance [9]. The crushing value, Los Angeles abrasion value, roundness, and flakiness index of coarse aggregates significantly influence the internal skeleton structure of SMA mixtures, which directly impacts their low- and high-temperature performance [10]. The mechanical properties of the SMA aggregate skeleton have been analysed using a three-dimensional spheroid model, with results indicating that increasing the number of contact points enhances the stability of the aggregate skeleton [11].

Therefore, determining the formation of the coarse aggregate skeleton structure in SMA mixtures is crucial for the effective design of the mixture. Brown [12] proposed the skeleton structure discrimination criterion for SMA mixture gradation design as *VCA*_mix_ < *VCA*_DRC_. *VCA*_DRC_ represents the percentage of voids in the coarse mineral aggregate under dry rodded conditions within the test vessel volume, whereas *VCA*_mix_ denotes the percentage of voids in the coarse mineral aggregate within the compacted SMA mixture. However, both *VCA*_mix_ and *VCA*_DRC_ values are influenced by factors such as testing methods, compaction procedures, and aggregate breakage. As a result, many researchers have questioned the reliability of using the *VCA*_mix_ < *VCA*_DRC_ criterion as a standard for identifying skeleton structure in SMA mixture gradation design [13]. In response, researchers have proposed alternative methods to determine whether the skeleton structure of coarse aggregates is properly formed in SMA mixtures. Miranda et al. [14] optimized the coarse aggregate structure of SMA mixtures using various laboratory compaction methods, including the manual dry–rodded method, standard Proctor compaction, modified Proctor compaction (light and heavy), and steel roller compaction. Among these, the Proctor and steel roller compactor methods, which are more representative of field conditions, are recommended for use in SMA mixture design. Meso-parameters, such as the average coordination number and the ratio of coarse aggregates without contact points to the total quantity of coarse aggregates, have been employed to ensure that the coarse aggregates interlock to form an optimal main skeleton [15]. Mixtures designed using this method exhibit excellent pavement performance, effectively improving the meso-parameters and significantly enhancing the quality of the main skeleton. The relationship between coarse aggregate gradation and controlling indices, such as VCA and CBR, was analysed by Zhang et al. [16], who recommended key aggregate sizes and particle size range contents for coarse aggregates. The results indicated a strong correlation between the coarse aggregate skeleton gradation of SMA mixtures and both VCA and CBR. Jiang et al. [17] proposed a reliable prediction model for VCA using a uniform design method and vibrating compaction tests, which aids in determining aggregate gradation during the design of SMA mixtures.

Although the aforementioned methods aid in identifying the skeleton structure of SMA mixtures, most rely on auxiliary measures to achieve this objective. Therefore, it is essential to develop simpler and more direct approaches for recommending the gradation of skeleton structure in SMA mixtures. However, compaction methods and compaction effort significantly influence the formation of the skeleton structure [18]. Consequently, given the increasing trend in pavement traffic characterized by “large flow, large-scale vehicles, heavy load, and overload,” the skeleton structure discrimination standard for SMA-13 mixtures should be revised to align with heavy-traffic compaction requirements [19].

Therefore, the primary objective of this research is to investigate the standard for discriminating skeleton structures in SMA-13 mixtures. This study presents the findings on factors influencing this standard. A *VCA*_mix_ calculation method based on the vibration compaction method (VTM) was proposed, and the effects of structural parameters on the high-temperature performance of SMA-13 mixtures were investigated. Finally, a standard for discriminating skeleton structures in SMA mixtures was recommended and validated based on the high-temperature performance of SMA-13 mixtures.

## 2. Materials and properties

### 2.1. Asphalt

SBS-modified asphalt sourced from Korea was used to prepare the SMA mixture in this study. The properties of the asphalt were tested [20] and are presented in Table 1.

**Table 1:** Properties of SBS modified asphalt.

Test items		Test results	JTG F40-2004 requirements [20]
Penetration (100 gramme, 5 s at 25 °C) (0.1 mm)		64.0	60 ~ 80
Ductility at 5^o^ C (cm)		41.2	30 minimum
Softening point, ^o^C (Ring & Ball Apparatus)		86.0	55 minimum
Density at 15 °C (g/cm^3^)		1.04	--
Reduction after RTFOT	Loss in mass (%)	−0.28	−1.0 ~ 1.0
	Penetration of residue (%)	81.4	60 minimum
	Ductility at 5^o^ C (cm)	26.1	20 minimum

### 2.2. Coarse aggregate

Basalt aggregate is generally used as the coarse aggregate in the SMA mixture. However, to compare the influence of different coarse aggregate types on the skeletal structure of the SMA mixture, limestone coarse aggregate was selected as a control group in this study. Coarse aggregates were sourced from three different quarries: Basalt aggregate-Ⅰ from Shangluo County, Shaanxi province; Limestone aggregate-Ⅰ from Liulin County, Shanxi province; and Basalt aggregate-Ⅱ from Luonan County, Shaanxi province. The properties of these coarse aggregates are listed in Table 2.

**Table 2:** Properties of coarse aggregates.

Test items	Test results of different sizes (mm) coarse aggregates						JTG F40-2004 requirements
	Basalt aggregate-Ⅰ		Limestone aggregate-Ⅰ		Basalt aggregate-Ⅱ		
	9.5 ~ 16	4.75 ~ 9.5	9.5 ~ 16	4.75 ~ 9.5	9.5 ~ 16	4.75 ~ 9.5	
Apparent specific gravity	2.921	2.930	2.722	2.731	2.901	2.903	2.6 minimum
Water absorption (%)	0.78	1.04	0.85	0.93	1.30	1.53	2.0 maximum
Flat and elongated particle in aggregate (%)	7.5	6.2	9.5	11.0	9.1	9.2	12 maximum
Aggregate impact value (%)	14.1		19.5		15.7		26 maximum
Los Angeles abrasion value (%)	15.3		22.5		15.0		28 maximum
Polished stone value	47		40		46		40 minimum
Adhesion grade to asphalt	5		5		5		5 minimum
Aggregate Soundness (%)	5.2		7.2		4.9		12 maximum

### 2.3. Fine aggregate

Three types of fine limestone aggregates were collected from Shangluo County, Liulin County, and Jingyang County. The properties of these fine aggregates are presented in Table 3.

**Table 3:** Properties of fine aggregates.

Test items	Test results of different fine aggregates			JTG F40-2004 requirements
	Fine aggregate-Ⅰ	Fine aggregate-Ⅱ	Fine aggregate-Ⅲ	
Apparent specific gravity	2.773	2.750	2.668	2.5 minimum
Aggregate Soundness (%)	4.7	4.6	3.1	12 maximum
Mud content (%)	0.3	0.2	1.0	3 maximum
Sand equivalent (%)	90	89.5	74.6	60 minimum
Methylene blue value (g/kg)	2.2	2.0	3.4	25 maximum
Angularity (s)	33	32.8	32.9	30 minimum

### 2.4. Mineral powder

Three types of mineral powders were collected from Shangluo County, Qishan County, and Jingyang County. The properties of these mineral powders are listed in Table 4.

**Table 4:** Properties of mineral powders.

Test items		Test results of different mineral powders			JTG F40-2004 requirements
		Mineral powder -Ⅰ	Mineral powder -Ⅱ	Mineral powder -Ⅲ	
Apparent specific gravity		2.782	2.771	2.649	≥2.5
Hydrophilicity coefficient		0.6	0.7	0.6	<1.0
Plasticity index		3.0	2.6	3.3	<4
Particle size range (%)	<0.6 mm	100	100	100	100
	<0.15 mm	98	96.5	99	90 ~ 100
	<0.075 mm	85	82.3	87.5	75 ~ 100

## 3. Influence factors of skeleton structure discrimination standard for SMA mixture

The prevailing viewpoint is that stone-on-stone contact between coarse aggregates ensures the SMA mixture exhibits strong resistance to loading deformation [21]. Moreover, the skeleton structure discrimination standard for SMA mixture gradation design is *VCA*_mix_ < *VCA*_DRC_, which requires verification under modern traffic conditions characterized by “large flow, large-scale vehicles, heavy load, and overload.” Therefore, this study recommends a standard for discriminating skeleton structures in SMA mixtures based on an analysis of the factors influencing the formation of the skeleton structure.

### 3.1 Influence of test method on the skeleton structure discrimination standard

*VCA*_DRC_, defined as the percentage of voids in the coarse mineral aggregate under dry-rodded conditions within the test vessel volume, was calculated using the Equation (1).


$${VCA}_{\text{DRC}}=(\text{1}-\frac{\gamma_{x}}{\gamma_{\text{ca}}})\times \text{100}$$ (1)

where *γ*_ca_ is the synthetic bulk specific gravity of coarse aggregates (g/cm^3^), *γ*_x_ is tamping accumulated density of coarse aggregates (g/cm^3^).

**Fig 1:**
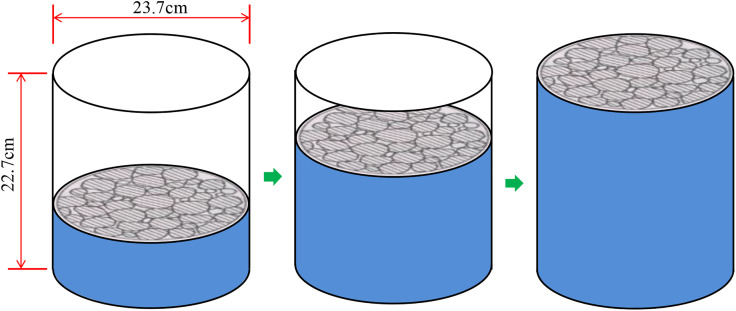
Schematic of dry rodded method.

*γ*_ca_ was determined using the mesh basket method [22], which includes the gross volume within the surface profile of the coarse aggregate, encompassing the aggregate entity and its open and closed pores. *γ*_x_ was determined using the dry-rodded method, in which the aggregate was loaded into a 10-L test vessel in three layers, with each layer tamped 25 times. A schematic of the dry rodded method is presented in Fig 1.

*VCA*_mix_, defined as the percentage of volume outside the coarse aggregate within the total volume of the asphalt mixture specimen, was calculated using the Equation (2).


$${VCA}_{\text{mix}}=(\text{1}-\frac{\gamma_{\text{f}}}{\gamma_{\text{ca}}}\times P_{\text{CA}})\times \text{100}$$ (2)

where *γ*_f_ is the bulk gravity of the asphalt mixture (g/cm^3^), *P*_CA_ is the percentage of coarse aggregates in the asphalt mixture (%).

**Fig 2:**
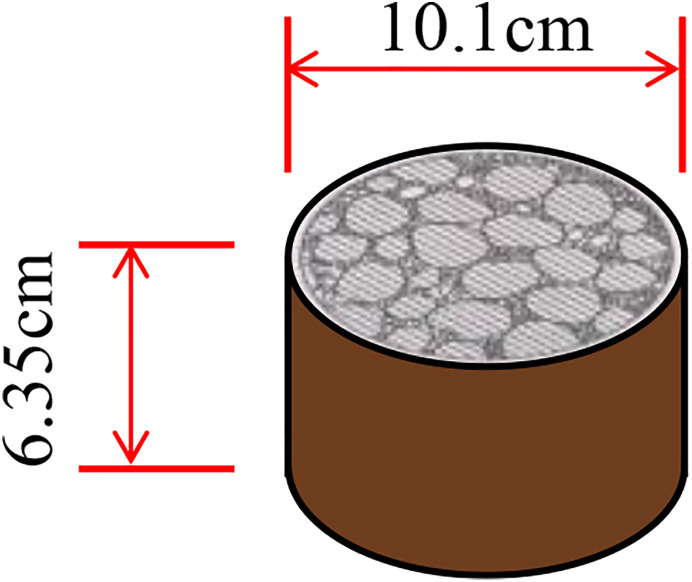
Schematic of SMA mixture specimen.

*γ*_f_ was determined using the surface-dry method, based on the test results of asphalt mixture specimens compacted in the laboratory. A schematic of the SMA mixture is shown in Fig 2.

A comparative analysis was conducted, revealing the following differences between the testing procedures and calculation methods.

The coarse aggregate was compacted by tamping with an iron rod to obtain the *VCA*_DRC_ value, which differs from the compaction effort applied during the preparation of the asphalt mixture specimen used to obtain the *VCA*_mix_ value. A 10,000 cm^3^ test vessel and a 515 cm^3^ specimen were utilized in the experimental procedures for determining *VCA*_DRC_ and *VCA*_mix_, respectively. These differing compaction volumes and methods affect the SMA-13 mixture, which has a nominal maximum particle size of 13.2 mm. The *VCA*_DRC_ calculation includes the surface open pores of the coarse aggregates, whereas the *VCA*_mix_ calculation does not account for the open pores in the asphalt mixture specimen. Consequently, the calculated *VCA*_DRC_ and *VCA*_mix_ values do not fully represent the actual values.

### 3.2. Influence of compaction method on the skeleton structure discrimination standard

**Fig 3:**
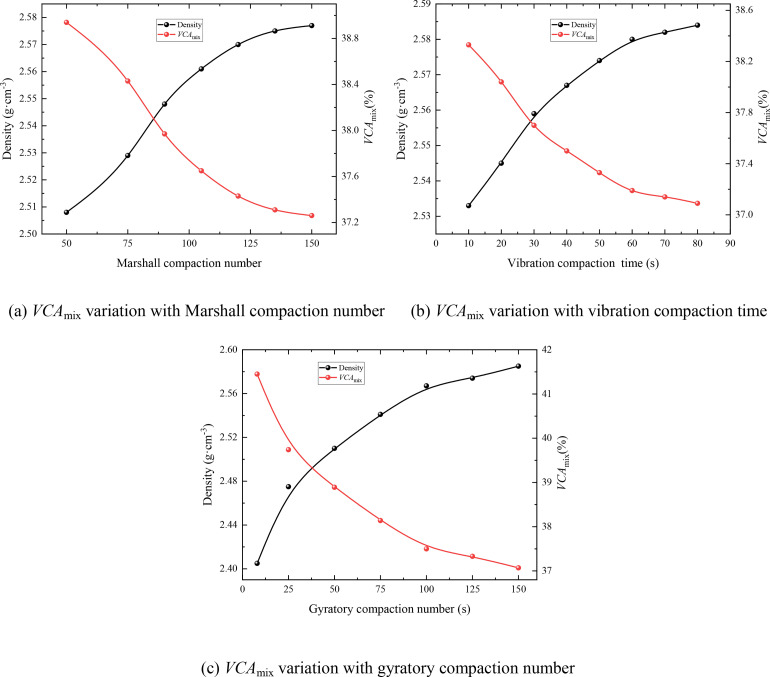
The effect of compaction number (time) on the *VCA*_mix_ of SMA mixture (a) VCAmix variation with Marshall compaction number (b) VCAmix variation with vibration compaction time (c) VCAmix variation with gyratory compaction number.

As shown in Equation 2, when the material and aggregate gradation are held constant, the calculated *VCA*_mix_ values correlate with the density of the SMA mixture specimen. SMA mixture specimens are typically prepared using the Marshall compaction, Superpave gyratory compactor, and vibration compaction methods (VTM) [23]. Accordingly, the relationship between the compaction method and the *VCA*_mix_ mixture was analyzed based on these compaction techniques. SMA mixtures with different compaction numbers (times) were prepared using gradation C and an asphalt aggregate ratio of 5.7%. The relationship between the compaction number (time) and *VCA*_mix_ for each compaction method is presented in Fig 3.

The *VCA*_mix_ of the SMA mixture decreased as the compaction number or compaction time increased under the same compaction method. Correspondingly, the density of the SMA mixture increased with compaction effort. These results are expected, as *VCA*_mix_ is inversely proportional to the density of the SMA mixture, as indicated by Equation 2. Therefore, different compaction methods and numbers (times) result in varying *VCA*_mix_ values for SMA mixtures [24]. These results may be attributed to the fact that, with a higher compaction number or time, the stone-on-stone contact among coarse aggregates in the SMA mixture becomes tighter, resulting in increased density and decreased *VCA*_mix_. Although the same materials are used, different compaction methods exhibit distinct compaction characteristics. In particular, the high-frequency excitation force applied during VTM compaction promotes interparticle movement, leading to a denser aggregate structure. Thus, the density of VTM-compacted specimens is higher than that of specimens prepared using other compaction methods.

### 3.3 Influence of aggregate breakage on the skeleton structure discrimination standard

**Fig 4:**
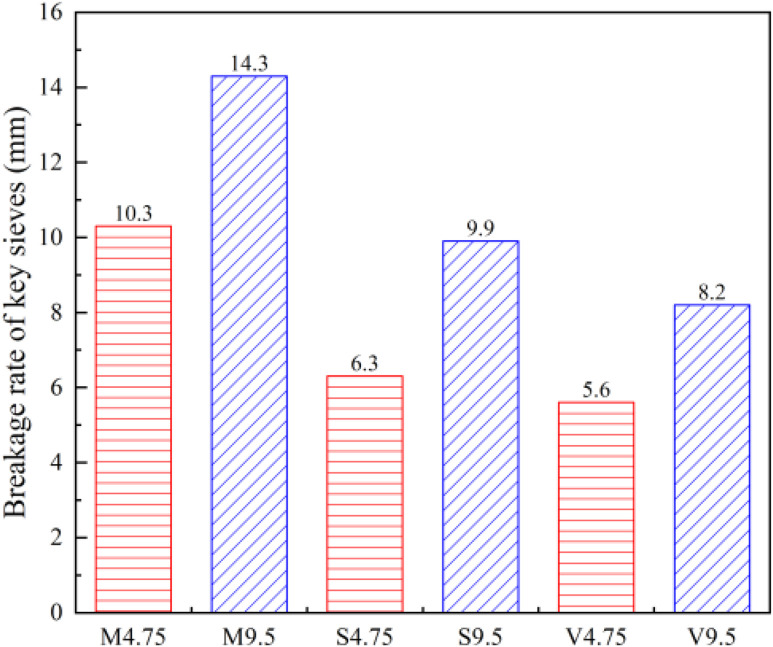
The breakage rate of key sieves of different compaction methods.

Several studies have demonstrated that aggregate breakage occurs during the compaction process of asphalt mixtures [25] and that both the compaction method and compaction effort influence the extent of aggregate breakage. The breakage rates of the SMA-13 mixture through key sieves (4.75 mm and 9.5 mm), using the various compaction methods (mentioned in Section 2.2), are presented in Fig 4. M4.75, S4.75, and V4.75 represent the breakage rates through the 4.75 mm sieve when the Marshall compaction method, Superpave gyratory compactor method, and VTM method were used, respectively. Similarly, M9.5, S9.5, and V9.5 represent the breakage rates through the 9.5 mm sieve for the respective compaction methods.

The results demonstrate that, under the same compaction effort, SMA-13 mixtures prepared using different compaction methods exhibit varying degrees of aggregate breakage through the key sieves. The Marshall compaction method results in the highest breakage rate, followed by the Superpave gyratory compactor method, with the VTM method exhibiting the lowest breakage rate. Furthermore, as shown in Fig 4, the breakage of aggregates larger than 4.75 mm is significantly lower than that of aggregates larger than 9.5 mm. This indicates that coarser aggregates are more susceptible to breakage during the compaction process.

Aggregate breakage affects not only the density of the SMA mixture, thereby influencing the *VCA*_mix_ calculation according to Equation 2, but also the overall performance of the SMA mixture. Therefore, selecting an appropriate compaction method for preparing SMA mixture specimens is crucial for obtaining accurate *VCA*_mix_ values and for reliably determining the skeleton structure of the SMA mixture [26]. Based on the results shown in Fig 4, the VTM method is recommended for designing SMA mixtures.

## 4. Calculation method of *VCA*_mix_ in SMA mixture based on the VTM method

The above discussion highlights that significant errors exist in both the experimental and mathematical determination of *VCA*_mix_ and *VCA*_DRC_. As a result, many researchers have questioned the validity of using *VCA*_mix_ < *VCA*_DRC_ as the skeleton structure discrimination standard for SMA mixture gradation design. Consequently, establishing a more reliable and reasonable standard for skeleton structure discrimination has become a major focus of research. As the asphalt mastic content in the SMA mixture decreases, the number of surface pores in the specimen increases after the skeleton structure is formed by the coarse aggregates. This relationship leads to a continuous decrease in the calculated value of *VCA*_mix_. Clearly, using Equation 2 to calculate changes in *VCA*_mix_ does not accurately reflect whether the skeleton structure of the SMA mixture has been formed. Therefore, this study introduces a volume-based term to modify the expression for *VCA*_mix_. The modified expression calculates the *VCA*_mix_ of the mixture specimen using the volume of the cylindrical specimen compacted by the VTM method. To distinguish it from the original expression in Equation (2), this study introduces the term *VCA*_v-mix_, which is expressed as Equation (3).


$${VCA}_{\text{v}-\text{mix}}=(\text{1}-\frac{m\times P_{\text{CA}}}{\text{π}\times \emptyset^{\text{2}}\times h}\times \frac{\text{1}}{\gamma_{\text{ca}}})\times \text{100}$$ (3)

where *P*_CA_ is the percentage of coarse aggregates in the asphalt mixture (%), *γ*_ca_ is the synthetic bulk specific gravity of the coarse aggregates (g/cm^3^), *m* is the mass of the SMA mixture specimen prepared using the VTM method (g), *h* is the height of the specimen (cm), *Φ* is the radius of the specimen (cm).

Equation (3) eliminates the influence of open voids in the SMA mixture when calculating the bulk specific gravity while accounting for the influences of compaction effort and method on the SMA mixture.

## 5. Influence of structural parameters on the high-temperature performance of SMA-13 mixture

To establish a reasonable skeleton structure discrimination standard for SMA mixtures, the structural parameters of the SMA-13 mixture with a skeleton-dense structure must be analysed. Research has shown that when the type of mineral aggregates remains consistent and the coarse aggregate gradation is constant, the *VCA*_mix_ value of the skeleton structure in the SMA mixture specimen remains unchanged. Accordingly, this study analysed the gradation and structural parameters (such as *VCA*_mix_, *VCA*_v*-*mix_, and *VCA*_DRC_) of SMA mixtures prepared with different types of mineral aggregates, as listed in Table 5, and investigated their influence on the shear strength and dynamic stability of the mixtures at high temperature. Based on this analysis, a standard for discriminating skeleton structures in SMA mixtures is proposed.

**Table 5:** Different mineral composition of SMA mixture.

Mixture types	Coarse aggregate	Fine aggregate	Mineral powder
Mixture-Ⅰ	Basalt aggregate-Ⅰ	Fine aggregate-Ⅰ	Mineral powder-Ⅰ
Mixture-Ⅱ	Limestone aggregate-Ⅰ	Fine aggregate-Ⅱ	Mineral powder-Ⅱ
Mixture-Ⅲ	Basalt aggregate-Ⅱ	Fine aggregate-Ⅲ	Mineral powder-Ⅲ

To investigate the influence of different mineral materials on the skeleton structure parameters of SMA mixtures, three types of SMA mixtures composed of mineral materials from different regions were selected, as listed in Table 5.

To examine the effect of varying 4.75 mm sieve passing rates on the skeleton structure parameters, five different gradations, as listed in Table 6, were adopted for each SMA mixture. The gradation of coarse aggregates (greater than 4.75 mm sieve) and fine aggregates (less than 4.75 mm sieve) for each gradation remained unchanged, whereas the ratio between coarse and fine aggregates (4.75 mm sieve passing rate) was varied. The cylindrical and track plate specimens were compacted using the VTM method under the optimal asphalt–aggregate ratio for each mixture. Then, the *VCA*_mix_, *VCA*_v-mix_, *τ*_d_, and *DS* were determined experimentally and analysed.

**Table 6:** The gradation of different mixture types.

Gruop	The passing rates (%) of different sieves (mm)								
13.2	9.5	4.75	2.36	1.18	0.6	0.3	0.15	0.075
1	100	59.8	34.0	26.8	21.8	18.2	15.8	14.0	10.0
2	100	58.0	31.0	24.7	20.3	17.2	15.0	13.5	10.0
3	100	56.2	28.0	22.6	18.8	16.2	14.3	13.0	10.0
4	100	54.4	25.0	20.5	17.4	15.1	13.6	12.5	10.0
5	100	52.6	22.0	18.4	15.9	14.1	12.9	12.0	10.0

**Fig 5:**
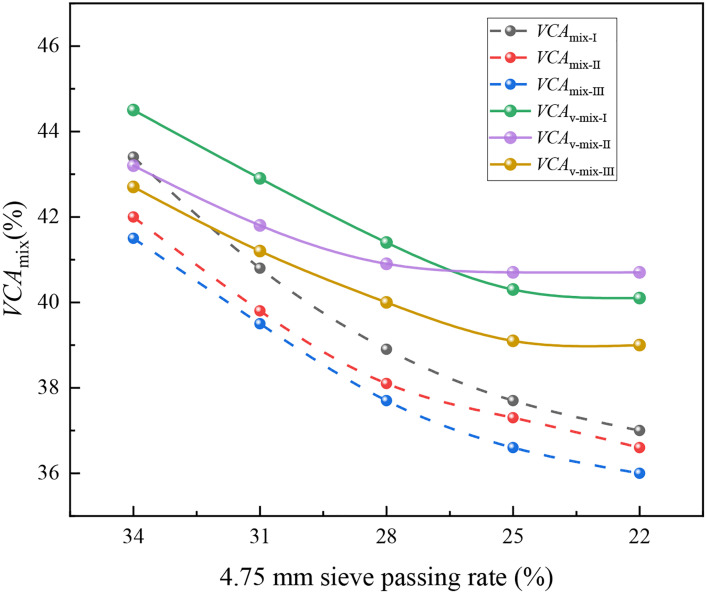
The *VCA*_mix_ of SMA mixture variation with different 4.75 mm sieve passing rate.

Fig 5 presents the variation of *VCA*_mix_ or *VCA*_v-mix_ in SMA mixtures with respect to the 4.75 mm sieve passing rate. The *VCA*_v-mix_ values were significantly greater than those of *VCA*_mix_, which accounts for the open voids in the SMA specimen when calculating the bulk specific gravity, as defined in Equation (3). For all SMA mixtures, *VCA*_mix_ decreased with a 4.75 mm sieve passing rate. In the case of mixtures I and III, which used basalt coarse aggregate, when the 4.75 mm sieve passing rate exceeded 25%, the *VCA*_v-mix_ value decreased with a reduction in the 4.75 mm sieve passing rate. However, when the 4.75 mm sieve passing rate was less than or equal to 25%, the *VCA*_v-mix_ value of the mixture remained approximately constant. For mixture II, which used limestone coarse aggregate, the *VCA*_v-mix_ value also remained stable when the 4.75 mm sieve passing rate was less than or equal to 28%. This confirms that the percentage of voids in the coarse mineral aggregate within the asphalt mixture of a skeleton-structured SMA specimen should remain unchanged. This phenomenon cannot be identified using the *VCA*_mix_ value but is clearly captured by the proposed *VCA*_v-mix_ value.

As shown in Fig 5, differences in skeleton-dense gradation were observed among SMA mixtures composed of different mineral aggregates. For mixtures I and III, the skeleton-dense gradation was achieved at a 4.75 mm sieve passing rate of 25%, whereas for mixture II, it was achieved at a 28% passing rate. This variation arises because the skeleton-dense structure of an SMA mixture is a spatial volume-based structure, whereas the 4.75 mm sieve passing rate reflects the mass ratio between coarse and fine aggregates. The densities of coarse and fine aggregates differ across various mineral sources, leading to differences in the passing rate at which the skeleton-dense structure is formed [21]. In other words, there is no universally applicable optimal skeleton-dense gradation for SMA mixtures with different mineral aggregates.

**Fig 6:**
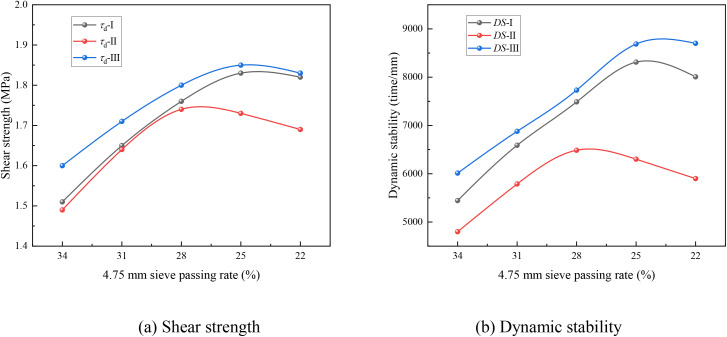
High temperature performance of SMA-13 mixture variation with 4.75 mm sieve passing rate (a) Shear strength (b) Dynamic stability.

To further verify that the skeleton-dense structure is formed in mixtures I and III at a 4.75 mm sieve passing rate of 25%, and in mixture II at a rate of 28%, the influence of the 4.75 mm sieve passing rate on the shear strength and dynamic stability of the SMA mixture was investigated. The results are presented in Fig 6.

As shown in Fig 6, the high-temperature performance of all SMA mixtures initially increased and then decreased as the 4.75 mm sieve passing rate decreased. This can be attributed to the fact that, at low sieve passing rates of 4.75 mm, the fine aggregate is insufficient to fill the voids between coarse aggregates, resulting in inadequate high-temperature performance of the SMA-13 mixture. As the 4.75 mm sieve passing rate increases, the voids within the coarse aggregate skeleton are gradually filled by fine aggregates, thereby enhancing the high-temperature performance. However, a further increase in the 4.75 mm sieve passing rate can disrupt the stone-on-stone contact within the coarse aggregate skeleton, leading to a reduction in high-temperature performance.

The 4.75 mm sieve passing rate corresponding to the maximum high-temperature performance aligns with the passing rate at which the *VCA*_v-mix_ value stabilizes. This confirms the formation of a skeleton-dense structure in mixtures I and III at a 4.75 mm sieve passing rate of 25%, and in mixture II at 28%, as verified by the high-temperature performance of the SMA mixtures. The values of *VCA*_mix-I_, *VCA*_mix-II_, and *VCA*_mix-III_ of the SMA mixtures with skeleton-dense structure gradation were 37.7%, 38.1%, and 36.6%, respectively. *VCA*_DRC-I_, *VCA*_DRC-II_, and *VCA*_DRC-III_ of SMA mixtures with each layer tamped 25 times during testing were 40.5%, 41.2%, and 39.5%, respectively. These results indicate that applying the criterion *VCA*_mix_ < *VCA*_DRC(n=25)_ as the skeleton structure discrimination standard for SMA mixture gradation design is unreasonable. Therefore, a more reliable standard for discriminating skeleton structures should be developed, particularly one suited to heavy-traffic compaction standards.

## 6. Skeleton structure discrimination standard for SMA mixture and its performance verification

As discussed in Section 2, insufficient tamping effort during the testing process is a key reason why the *VCA*_mix_ value of an SMA mixture was significantly lower than its corresponding *VCA*_DRC_ value. The standard practice of tamping each layer 25 times, as specified in test protocols, results in a compaction effort that is considerably lower than that applied during specimen compaction, thereby limiting its effect on the coarse aggregate structure. However, applying a compaction effort similar to that used in specimen preparation may cause aggregate breakage owing to the lack of the lubricating effect provided by asphalt binder, thus affecting the accuracy of the experimental *VCA*_DRC_ results. To more accurately establish a skeleton structure discrimination standard for SMA mixtures, *VCA*_DRC_ was evaluated under varying rod tamping times. The gradation ratio of coarse (9.5–13.2 mm) to fine (4.75–9.5 mm) aggregates was maintained at 60:40. The *VCA*_DRC_ values corresponding to different tamping times per layer are listed in Table 7.

**Table 7:** *VCA*_DRC_ value with different tampe times.

Coarse aggregate types	Tampe times of each layer (n)					
25	50	75	100	125	150
Basalt aggregate-Ⅰ	40.5	40	39.7	39.5	39.3	39.3
Limestone aggregate-Ⅰ	41.2	40.7	40.4	40.2	40.0	39.9
Basalt aggregate-Ⅱ	39.5	39	38.8	38.6	38.5	38.5

**Fig 7:**
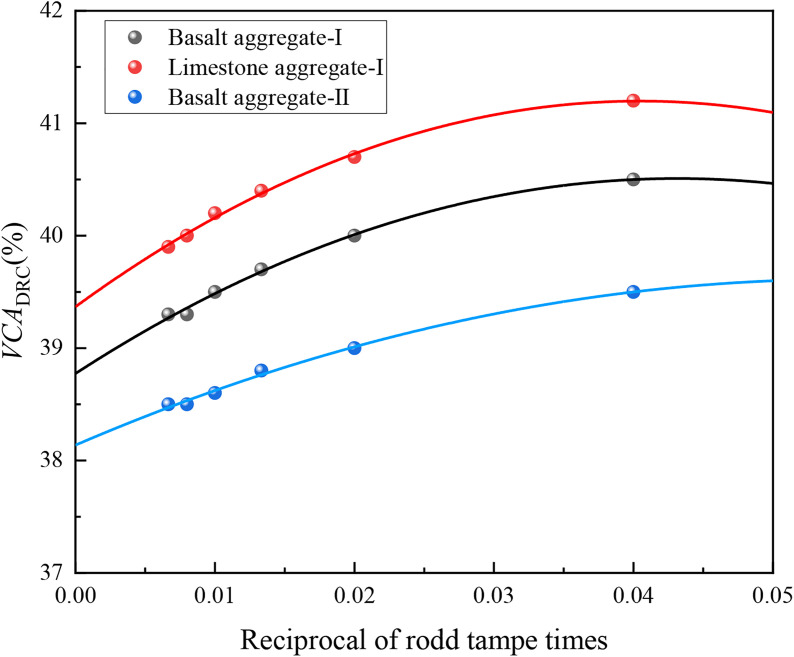
*VCA*_DRC_ value of different mixtures variation with the tampe times.

As shown in Table 7, the *VCA*_DRC_ value decreased gradually with an increase in rod tamping times per layer, but the amplitude decreased. To determine the limiting value of *VCA*_DRC_, let *x* = 1/n, and *x* = 0 when the number of rod tamping times is n → ∞. The relationship between *VCA*_DRC_ and rod tamping times is shown in Fig 7.

According to the fitting results in Fig 7, the *VCA*_DRC(n→∞)-I_, *VCA*_DRC(n→∞)-II_, and *VCA*_DRC(n→∞)-III_ values of the SMA mixture were 38.8%, 39.4%, and 38.1%, respectively, which are 4%–5% lower than the *VCA*_DRC(n=25)_ values for all coarse aggregates. Considering that the calculation of *VCA*_DRC(n→∞)_ value is too cumbersome, *VCA*_DRC(n→∞)_ was taken as 0.95 *VCA*_DRC(n=25)_. The different void indices of coarse aggregates or mixtures are listed in Table 8.

**Table 8:** Different voids index of coarse aggregates or mixture.

Mixture types	*VCA*_mix_ with skeleton dense structure gradation	*VCA*_DRC_	0.95*VCA*_DRC_
Mixture-Ⅰ	37.7	40.5	38.5
Mixture-Ⅱ	38.1	41.2	39.1
Mixture-Ⅲ	36.6	39.5	37.5

As shown in Table 8, for the SMA-13 mixture with skeleton-dense structure gradation, the *VCA*_mix_ value was significantly lower than the *VCA*_DRC_ value, indicating that the *VCA*_DRC_ value calculated using compacted coarse aggregates tamped with an iron rod cannot accurately reflect the skeleton-dense aggregate structure of the SMA mixture. Furthermore, the *VCA*_mix_ value with skeleton-dense structure gradation was less than 0.95*VCA*_DRC(n=25)_. This is because the calculation process did not account for the surface-opening voids of the mixture specimens, thereby reducing the calculated *VCA*_mix_ value [27]. Moreover, aggregate particles may undergo slight crushing during the vibration compaction method, which results in a *VCA*_mix_ value smaller than the *VCA*_DRC(n→∞)_ value for the SMA-13 mixture with skeleton-dense structure gradation. However, 0.95*VCA*_DRC_ is approximately equal to the calculated *VCA*_mix_ value of the SMA-13 mixture with skeleton dense-structure gradation.

**Fig 8:**
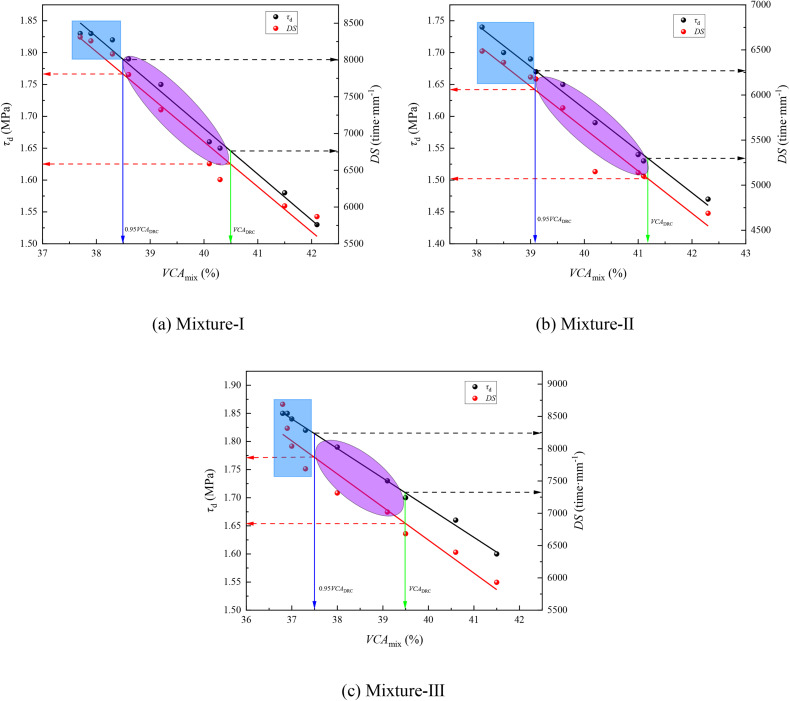
High temperature performance of different SMA-13 mixtures variation with *VCA*_mix_ value (a) Mixture-Ⅰ (b) Mixture-Ⅱ (c) Mixture-Ⅲ.

To further illustrate the influence of the skeleton structure discrimination standard on the high-temperature performance of designed SMA mixtures, the relationship between *VCA*_mix_ and the high-temperature performance of different types of SMA-13 mixtures is shown in Fig 8.

Fig 8 shows that when the skeleton structure of the SMA mixtures was still forming (*VCA*_mix_ > *VCA*_DRC(n=25)_), the shear strength and dynamic stability of the SMA mixtures exhibited an increasing trend as the *VCA*_mix_ of the SMA mixtures decreased. This can be explained as follows: when the mineral type and gradation of the SMA mixture are fixed, that is, the proportion of coarse aggregates and the synthetic bulk specific gravity of coarse aggregates are determined, then, according to Equation 2, as the bulk gravity of the SMA mixture specimen increases, the *VCA*_mix_ decreases. Therefore, as the *VCA*_mix_ decreases, the aggregates rearrange to form a more compact skeleton structure, leading to an increase in the bulk gravity of the specimen and consequently, an improvement in the high-temperature performance (such as shear strength and dynamic stability) of the SMA mixture.

Compared with the SMA mixtures designed using *VCA*_mix_ < *VCA*_DRC(n=25)_ as the skeleton structure discrimination standard, those designed using *VCA*_mix_ < 0.95*VCA*_DRC(n=25)_ exhibited more prominent high-temperature performance. Especially, when *VCA*_mix_ < *VCA*_DRC(n=25)_ was adopted as the discrimination criterion, the performance of the designed SMA mixtures tended to be lower—particularly in the elliptical area shown in Fig 8—compared to those designed using *VCA*_mix_ < 0.95*VCA*_DRC(n=25)_, which fall within the rectangular area of Fig 8. Therefore, *VCA*_mix_ < 0.95*VCA*_DRC(n=25)_ is recommended as the skeleton structure discrimination standard for SMA mixture gradation design to improve the high-temperature performance of the resulting mixtures.

The shear strength and dynamic stability of SMA-13 mixtures with *VCA*_mix_ = *VCA*_DRC(n=25)_ and 0.95*VCA*_DRC(n=25)_ were obtained to evaluate the applicability of the skeleton structure discrimination standards *VCA*_mix_ < *VCA*_DRC(n=25)_ and *VCA*_mix_ < 0.95*VCA*_DRC(n=25)_. The comparative results of the performance characteristics of the different SMA-13 mixtures are listed in Table 9.

**Table 9:** Performance comparison results of different SMA-13 mixtures.

Mixture types	*VCA*_mix_ = *VCA*_DRC_		*VCA*_mix_ = 0.95*VCA*_DRC_	
	τ_d-1_/τ_max_	*DS*_-1_/*DS*_max_	*τ*_d-2_/*τ*_max_	*DS*_-2_/*DS*_max_
Mixture-Ⅰ	0.90	0.79	0.98	0.94
Mixture-Ⅱ	0.88	0.78	0.96	0.94
Mixture-Ⅲ	0.92	0.79	0.98	0.90
Average value	0.90	0.79	0.97	0.93

The *τ*_max_ is the peak shear strength of SMA-13 mixture, and the *DS*_max_ is the peak dynamic stability of SMA-13 mixture.

As shown in Table 9, when *VCA*_mix_ = 0.95*VCA*_DRC(n=25)_, the high-temperature performance of the SMA-13 mixture approached its peak strength, indicating that the skeleton structure of the SMA mixture is most stable under this condition. The average shear strength and dynamic stability of the SMA-13 mixture reached 97% and 93% of their respective peak strength values. By contrast, when *VCA*_mix_ = *VCA*_DRC(n=25)_, the average shear strength and dynamic stability of the SMA-13 mixture were 90% and 79% of their peak strength values, respectively. As the *VCA*_mix_ value of the SMA mixture increased, the shear strength and dynamic stability of the mixture decreased. Hence, *VCA*_mix_ < 0.95*VCA*_DRC(n=25)_ is considered an appropriate skeleton structure discrimination standard for SMA mixture gradation design, as it enables the production of mixtures that exhibit performance characteristics closer to the optimal values [28,29]. Accordingly, *VCA*_mix_ < 0.95*VCA*_DRC(n=25)_ is recommended as the skeleton structure discrimination standard for SMA mixture gradation design.

During the SMA mix design process, three to five trial aggregate gradations with varying mineral compositions are initially selected. The *VCA*_DRC_ and *VCA*_mix_ of different gradations are calculated based on the cylindrical specimen compacted by the VTM method. The optimal gradation is then selected based on the skeleton structure discrimination standard (*VCA*_mix_ < 0.95*VCA*_DRC_), ensuring that the SMA mixture achieves a interlocked coarse aggregate skeleton. Such a design philosophy significantly enhances the mixture’s high-temperature stability, rendering it particularly suitable for demanding applications in heavy-duty asphalt pavements where superior rutting resistance and durability are paramount.

## 7. Summary and conclusions

In this study, the influence of structural parameters on the high-temperature performance of an SMA-13 mixture was analyzed, and a standard for discriminating skeleton structures in SMA mixtures was recommended and validated based on the obtained results. The main conclusions of this study are as follows:

The factors influencing the standard for discriminating skeleton structures in SMA mixtures—such as the test method, compaction method, and aggregate breakage—were thoroughly analysed. It was determined that variations in testing methods, compaction efforts, and coarse aggregate breakage contribute to inconsistencies between the key parameters, *VCA*_DRC_ and *VCA*_mix_, when assessing the skeleton structure discrimination standard for SMA mixtures.

The *VCA*_v-mix_ expression, which accounts for the influence of open voids in the SMA mixture specimens, as well as compaction effort and method, is proposed to obtain an SMA mixture with a dense skeleton structure.

The gradation and structural parameters (such as *VCA*_mix_, *VCA*_v*-*mix_, and *VCA*_DRC_) of SMA mixtures with different types of mineral aggregates were examined, and their effects on the shear strength and dynamic stability of the mixtures at high temperature were analysed. Differences were observed in the skeleton-dense gradation of SMA mixtures prepared with different mineral aggregates. The skeleton-dense gradation of mixtures I and III was formed when the 4.75 mm sieve passing rate was 25%, whereas that of mixture II was formed when the 4.75 mm sieve passing rate was 28%.

The influence of tamping times per layer on the *VCA*_DRC_ value of coarse aggregates was investigated. The results indicated that the *VCA*_DRC_ value decreased with increasing rod tamping but eventually tended toward stabilization. Based on the fitting curve analysis, the *VCA*_DRC(n→∞)_ value was found to be approximately equivalent to 0.95 *VCA*_DRC(n=25)_.

Therefore, the condition *VCA*_mix_ < 0.95*VCA*_DRC(n=25)_ is recommended as the skeleton structure discrimination standard for SMA mixture gradation design, as it enables the attainment of an SMA mixture with performance characteristics closer to the optimum.
